# Unidirectional invisibility induced by parity-time symmetric circuit

**DOI:** 10.1038/srep40575

**Published:** 2017-01-18

**Authors:** Bo Lv, Jiahui Fu, Bian Wu, Rujiang Li, Qingsheng Zeng, Xinhua Yin, Qun Wu, Lei Gao, Wan Chen, Zhefei Wang, Zhiming Liang, Ao Li, Ruyu Ma

**Affiliations:** 1 Microwave and Electromagnetic Laboratory, Harbin Institute of Technology, No. 92, Xidazhi Street, Nangang District, Harbin City, Heilongjiang Province, China; 2 School of Electronic Engineering, Xidian University, Xi’an, 710071, China; 3 College of Information Science and Electronic Engineering, Zhejiang University, Hangzhou 310027, China; 4 Propagation Group, Wireless Technologies Branch, Communications Research Centre Canada, Government of Canada, 3701 Carling Ave., Box 11490, Station H, Ottawa, Ontario K2H 8S2, Canada; 5 Harbin medical university, No. 157, Baojian Street, Nangang District, Harbin City, Heilongjiang Province, China

## Abstract

Parity-time (PT) symmetric structures present the unidirectional invisibility at the spontaneous PT-symmetry breaking point. In this paper, we propose a PT-symmetric circuit consisting of a resistor and a microwave tunnel diode (TD) which represent the attenuation and amplification, respectively. Based on the scattering matrix method, the circuit can exhibit an ideal unidirectional performance at the spontaneous PT-symmetry breaking point by tuning the transmission lines between the lumped elements. Additionally, the resistance of the reactance component can alter the bandwidth of the unidirectional invisibility flexibly. Furthermore, the electromagnetic simulation for the proposed circuit validates the unidirectional invisibility and the synchronization with the input energy well. Our work not only provides an unidirectional invisible circuit based on PT-symmetry, but also proposes a potential solution for the extremely selective filter or cloaking applications.

PT-symmetry presents an interesting performance that the Hamiltonian has the entirely real-energy spectrum below the phase transition point, which can be extended to quantum theory^1^,^2^,^3^,^4^. Recently, the PT-symmetric theory in quantum mechanics is introduced to optical field^5^. The classical systems consisting of amplifier^6^, photon^7^ or periodic nanostructure^8^,^9^,^10^ lead to the achievement of the PT-symmetry in the optical frequencies, which has open up a new perspective towards achieving the optical waveguide^6^, power oscillation^8^,^11^, loss-induced transparency^12^, nonreciprocal Bloch oscillations^13^, laser absorber^14^, unidirectional invisibility^15^,^16^,^17^, and various extraordinary nonlinear effects^18^,^19^,^20^. Furthermore, the PT-symmetry can be used in the optical device, e. g. the optical-locking component^21^ and promises applications in electric and acoustic field additionally^22^,^23^.

In optics, the striking performance of the PT-symmetry is the unidirectional invisibility at the spontaneous PT-symmetry breaking point^15^,^16^,^17^. The optical systems present reflectionless around the Bragg resonance at the one side, and enhanced reflectivity from the other side contrarily^15^. Furthermore, when the lossy and active sheets are inserted in front and behind the almost fully reflective sheets, the incident waves are replicated behind the PT-symmetric structure in synchronization with the input signal^24^. Based on the effective-mapping image between the quantum and the classical mechanism, the circuit and the microwave system can be devoted to the PT-symmetry devices in theory and experiment^25^,^26^. In the electric system, the lumped resistor and amplifier are analogous to the attenuation and amplification in the complex potential of Hamiltonian operator.

In this paper, we formulate the PT-symmetric circuit consisting a resistor and a microwave TD with the negative impedance which represent the attenuation and amplification. Based on the scattering matrix method, the circuit presents extremely weak reflection at one side and enhanced reflection from the other side respectively. Additionally, the bandwidth of the unidirectional invisibility can be tuned by the resistance of the reactance element. Furthermore, the electromagnetic simulation validates the unidirectional invisibility and the “teleport” performance in the proposed circuit. Our work provides the PT-symmetry in electric circuit and opens up the possibilities to construct the extremely selective filter and electric-cloaking applications.

## Results

**Figure 1:**
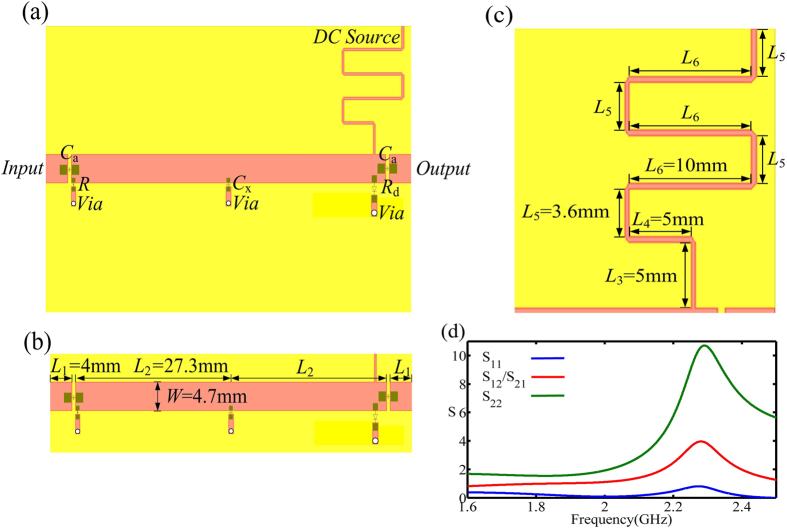
The layout of the PT-symmetric circuit. (**a**) The added capacitor *C*_*a*_ = 1*μF* embedded in package 0805, and the capacitor *C*_*x*_ = 4*pF* embedded in package 0603. The resistor *R* = 50 Ω with package 0603 and the negative *R*_*d*_ = −50 Ω represents the TD. (**b**) The size of the main thread. (**c**) The size of the DC-source supply system of the TD261A. (**d**) The S-parameter of layout simulation. The amplitude of *S*_11_ approches to zero from the input, and the amplitude of *S*_22_ is higher than unitary which leads to the reflection at the output. Additionally, the amplitude of *S*_12_/*S*_21_ is approximate unitary which illustrates that the full transmission of the circuit.

The layout of the PT-symmetric circuit is shown in Fig. 1. The added capacitors *C*_*a*_ = 1 *μF* with package 0805 are placed on the both sides of the circuit for DC-blocking. In addition, the strip line *L*_1_ = 4 mm is the platform of the external interface. The capacitor *C*_x_ = 4 pF with package 0603 is parallel in the middle of the main thread, and the distance between *C*_x_ and *C*_a_ is *L*_2_ = *λ*/4 = 27.3 *mm*. The resistor *R = *50 Ω with package 0603 is laid parallel in the input, and the tunnel diode TD261A with the negative resistor *R*_*d*_* = −*50 Ω is parallel in the output. The width of the strip line is *W* = 4.7 mm for the characteristic impedance *Z*_0_* = *50 Ω, and the via connects the electric components to the background of the circuit board, as shown in Fig. 1(b). The DC-source system of the TD261A is implemented at the output of the main thread. The length and the width of the serpentine line is *L = L*_3_ + *L*_4_ + 4*L*_5_ + 3*L*_6_ = *λ*/2 = 55.6 *mm* and 0.4 mm for blocking the AC signal on the main thread and flowing the DC energy effectively, as shown in Fig. 1(c). Under the perfect reflection of the reactance component, the attractive performance of the proposed circuit presents replicating the input signal in synchronization behind the structure^24^. More specifically, at the spontaneous PT-symmetry breaking point, the reflection from one end of the circuit is diminished while it is enhanced from the other, and the transmission coefficient is nearly unitary which presents the unidirectional invisibility, as shown in Fig. 1(d).

### Theory

**Figure 2:**
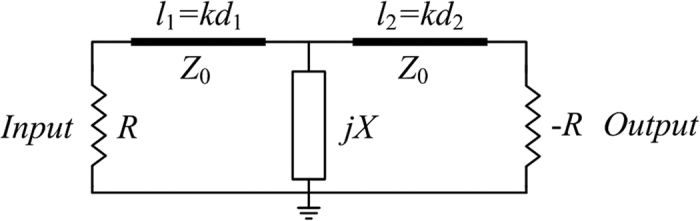
The schematic model of the electric PT-symmetric circuit. The main-energy stream flows from the lumped resistor *R* to the active element -*R* and the reactance component is represented by *jX*. The model of transmission lines is indicated by the electric length *l*_1 _= *kd*_1_, *l*_2 _= *kd*_2_ and characteristic impedance *Z*_0_, where *k* is the vector number and *d*_1_, *d*_2_ is the physical length of lines.

We formulate the PT-symmetric circuit consisting of the lumped elements and the microstrip line, as shown in Fig. 2. The negative resistor is implemented by the TD which possesses the relation *U*_d_ = −*IR*_d_^27^, and the loss performance is realized by the lumped resistor *R*. Here we regulate the voltage *U*_d_ across the TD to realize the balanced-resistance relation *R*_d_ = *R* for PT-symmetric distribution. The two parallel resistors are separated by two transmission lines of which the electric lengths are *l*_1_ = *kd*_1_, *l*_2_ = *kd*_2_ and the characteristic impedance is *Z*_0_, in which *k* is the wave number and *d*_1,2_ is the physical lengths of the transmission lines. Furthermore, the resistance of reactance component which consists the capacitor *C* or the inductor *L* is *X* = 1/*ωC* or *X* = *ωL* between the two transmission lines. Here we note *r* and *x* which represent the normalized resistances *r* *= R/Z*_0_ and *x* *= X/Z*_0_. In order to eliminate the reflection at port 1, the input impedance is consistent with the characteristic impedance of the transmission lines that is *R* *= Z*_0_ and *r* = 1. The scattering matrix of the electric circuit can be calculated by the transmission-matrix approach (Supplementary Note 1):


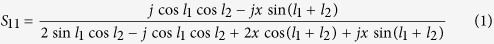



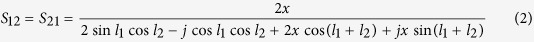



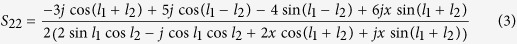


It is straightforward to verify that the *S* matrix fulfills the PT-symmetry formation PTS(ω^*^)*PT* = *S*^*−*1^(*ω*)^28^. When the lengths of the transmission lines satisfy *l*_1_, *l*_2_ = *π*/2 + *Nπ* where *N* is positive integer, the scattering matrix is:


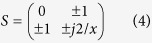


Additionally, the eigenvalues of the S-matrix at this condition are calculated as:


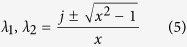


In Eq. 4, the reflection from the input *S*_11_ is zero and the transmissions *S*_12_ and *S*_21_ are unitary independent of *x*. When the resistance of the reactance component is far less than the characteristic impedance *Z*_0_ that represents the normalized relation *x* ≪ 1, the eigenvalues of the scattering matrix are pure imaginary and the reflection from the output *S*_22_ ≫ 2 presents the highly enhanced reflection than unitary. Furthermore, the unidirectional invisibility is shown in the circuit at this condition accordingly. When the resistance of the reactance component is equal to *Z*_0_ that satisfies the normalized relation *x* = 1, the magnitude of the reflection from the output is 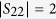 and the eigenvalues of the scattering matrix are 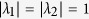 at the spontaneous PT-symmetry breaking point. When the resistance of the reactance component is greater than *Z*_0_ that presents normalized relation *x *≫ 1, the eigenvalues of the scattering matrix changes to complex from the pure imaginary and the magnitude of reflection 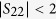 which leads to the electric circuit system presenting poorly unidirectional performance. Therefore, the resistance of the reactance component can tune the unidirectional states of the proposed circuit, and the normalized relation *x* = 1 is corresponding to the spontaneous PT-symmetry breaking point which is the watershed of the unidirectional invisibility.

A condition of special interest is given by *l*_1_ = *l*_2_ → *π*/2, for which we obtain the approximation relations that are 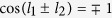, 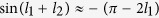, and the amplitude of scattering matrix can be calculated as:


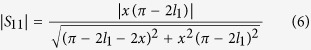



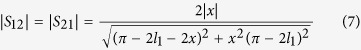



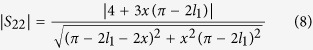


When the electric lengths of the two transmission lines are *l*_1_ = *l*_2_ which satisfy the phase relation *π* − 2*l*_1_ = 2*x* for fixed positive value *x* and *d*_1_, we can get the special frequency from the electric lengths expression *l*_1_ = *l*_2_ = *kd*_1_ = 2*πfd*_1_/*v*.


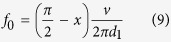


**Figure 3:**
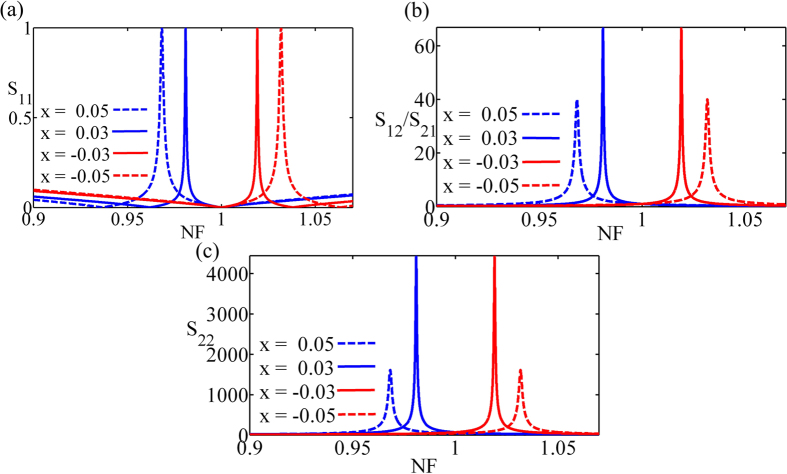
The scattering performance of the electric PT-symmetric circuit. (**a**) The amplitude of reflection *S*_11_ from the input presenting unitary at various normalized quantities of reactance component *x*. (**b**) The transmission amplitude inversely proportion to the value of *x*. (**c**) The amplitude of the reflection *S*_22_ from the output presenting inversely proportion to the value of *x*. Here the normalized frequency (NF) represents that the frequency is normalized by the the transparent frequency which corresponds to the phase relation *l*_1_ = *l*_2_ = *π*/2.

Here the propagation velocity *v* is determined by the physical condition of transmission line, which is implemented by microstrip-line formation. For the smaller positive value *x* that is *x* ≪ 1, the specific frequency *f*_0_ is slightly lower than the transparent frequency which is corresponding to the *l*_1_ = *l*_2_ = *π*/2. When the value of normalized quantities of reactance component *x* is negative, the specific frequency *f*_0_ is slightly higher than the transparent frequency respectively. In Eq. (6, 8), it is remarkable that the reflection amplitudes of input is fixed unitary at various specific frequencies, and the reflection amplitudes of output is far larger than unitary due to the numerator and the denominator presenting approximation constant and minimal value close to zero which is inversely proportional to the value of *x*. The amplitudes of transmission 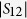 and 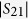 expressed as 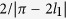 at the special frequency *f*_0_ are larger than unitary because the electric length *l*_1_ → *π*/2, and present inversely proportion to the value of *x* due to the phase relation *π* − 2*l*_1_ = 2*x*. Additionally, when the value of *x* is smaller, the special frequency *f*_0_ closes to the transparent frequency corresponding to the phase relation *l*_1_ = *l*_2_ = *π*/2. Based on the above theory, we format the electric circuit consisting of the lumped elements and ideal transmission lines in which the the propagation velocity is speed of light in vacuum *v* = 3 × 10^8^ *m/s*, and the frequency is normalized by the the transparent frequency. With the various normalized quantities of reactance component *x* = 0.05, 0.03, −0.03, −0.05, the special-normalized frequencies are 0.9682, 0.9809, 1.0191, 1.0318 which distribute symmetrically around the normalized-transparent frequency *f* = 1. Furthermore, the reflection amplitudes of input at the special frequencies are constant unitary, as shown in Fig. 3(a). The reflection amplitudes of port 2 at the special frequencies 0.9682 and 0.9809 are 1630 and 4447.4, which presents that the reflection amplitude of output are inversely proportional to the value of *x*, and the reflection at the special frequencies 1.0191 and 1.0318 are mirror symmetry with the lower frequencies, as shown in Fig. 3(c). The scattering-transmission amplitudes are 40 and 66.7 at the special frequencies 0.9682 and 0.9809, which presents the transmission amplitude are inversely proportional to the value of *x*, and the transmission performance at the special frequencies 1.0191 and 1.0318 are mirror symmetry with the lower frequencies, as shown in Fig. 3(b).

### Electromagnetic simulation

Based on the above theory, we tune the scattering performance of the PT-symmetric circuit by the mcrostrip lines between the electric elements, which are constructed based on the 1.524-mm-thick Rogers-5880 substrate whose dielectric constant is *ε*_*r*_ = 2.2. From the equivalently replacing the air and dielectric regions by a homogeneous medium, the effective dielectric constant of the microstrip line is given approximately by


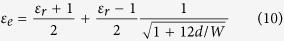


where *W* and *d* represent the microstrip-line width and the substrate height. Additionally, the *W/d* ratio can be found as


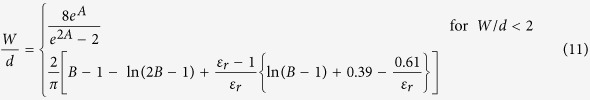


where 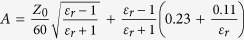 and 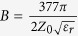.

For a given characteristic impedance *Z*_0_ = 50 Ω, *d* = 1.524 mm and the substrate dielectric constant *ε*_*r*_ = 2.2, we can get the microstrip-line width *W* = 4.7 *mm*. From the phase velocity 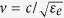 and propagation constant 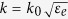, the length of microstrip line is *d*_1_ = *d*_2_ = 27.3 mm corresponding to the phase relation *l*_1_ = *l*_2_ = *π*/2^29^.

**Figure 4:**
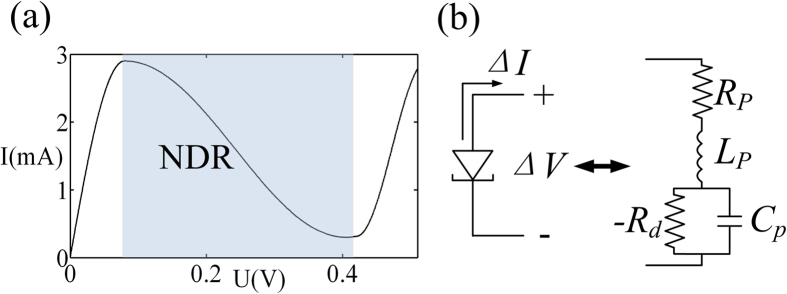
The equivalent circuit of the TD261A and its I-V curve. (**a**) The grey area indicates the NDR region, the negative resistance -*R*_d_ is from −50 Ω to 250 Ω^27^. (**b**) The passive elements *R*_p_, *L*_p_ and *C*_p_ are the parasitic resistance, inductance and capacitance of the TD261A respectively.

The negative differential resistance (NDR) at microwave frequencies is offered by the quantum tunnelling semiconductor devices, which is realized by General Electric’s TD261A^30^,^31^. The NDR can be equivalently considered as a negative resistance −*R*_d_, acting as a current source linearly controlled by an applied voltage which is shown as in Fig. 4(a). Furthermore, the equivalent circuit of the TD261A is provided in Fig. 4(b). In the NDR region, TD261A is composed of a negative resistance −*R*_d_ and the parasitic components *R*_p_, *L*_p_ and *C*_p_ representing the device package. Although the high-frequency limit of TD261A can be up to 26 GHz, to minimize the impact of the parasitic parameters, the working frequency of the PT-symmetric circuit is chosen to be around 2 GHz. By the bias voltage of 0.38 V, the TD261A parameters are: *R*_*p*_ = 7 Ω, *L*_*p*_ = 1.5 *nH, C*_*p*_ = 0.65 *pF* and −*R*_*d*_ = −50 Ω. Furthermore, we add the capacitors *C*_*a*_ = 1*μF* at the front and behind the main thread of the circuit and validate that *C*_a_ has little effect on the AC state of the circuit (Supplementary Note 3).

**Figure 5:**
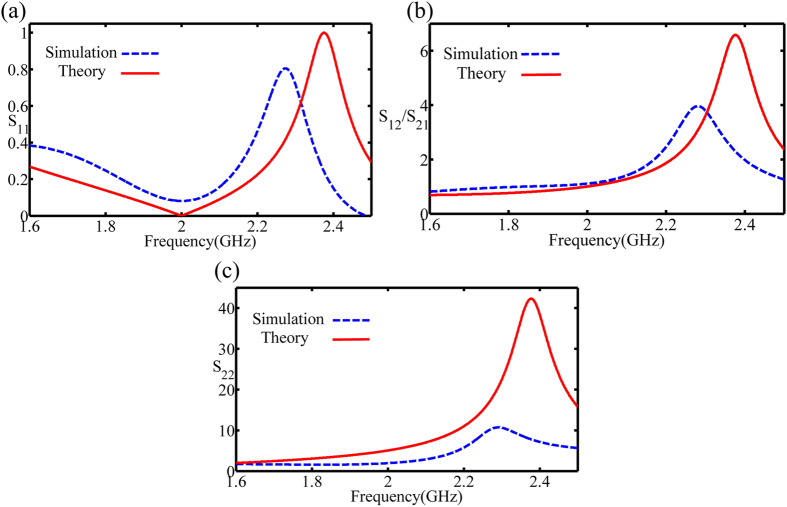
The comparison of the scattering matrix in theory and layout simulation. (**a**) The reflection *S*_11_ from the input. (**b**) The transmission *S*_12_/*S*_21_. (**c**) The reflection *S*_22_ from the output. The deviation in the theory and simulation is due to the substrate and copper loss.

Here the layout simulation is carried out using the commericial electromagnetic software package (CST Microwave Studio). The *S*-parameter comparison of the theory and the layout simulation with the reactance component *C*_x_ = 4 pF is shown in Fig. 5. The amplitude of *S*_11_ representing the reflection from the input is about 0.1 at 2 GHz due to the influence of the equivalent parasitic capacitance and inductor of the input-resistance package, as shown in Fig. 5(a). The amplitude of *S*_12_/*S*_21_ is about 0.9 at 2 GHz presenting a nonideal transmitted state because of the loss of the dielectric substrate and the copper. The parameter *S*_22_ is about 1.9 which presents a strong reflection and thus a well-defined unidirectional performance, as shown in Fig. 5(c).

## Discussion

In conclusion, we formulate the PT-symmetric circuit consisting of the loss-component resistor and the amplified TD261A. Additionally, we insert the lumped capacitor as the fully reflective element parallel between the two transmission lines. Layout simulations show that the circuit exhibits a unidirectional invisibility. Based on the scattering matrix theory, we illustrate that the circuit presents an extremely weak reflection from input and a stronger reflection than unitary from output respectively. Besides, the proposed-parity PT-symmetric circuit opens up an electric unidirectional aspect and provides an extremely selective filter and electric-cloaking applications.

## Additional Information

**How to cite this article**: Lv, B. *et al*. Unidirectional invisibility induced by parity-time symmetric circuit. *Sci. Rep.* **7**, 40575; doi: 10.1038/srep40575 (2017).

**Publisher's note:** Springer Nature remains neutral with regard to jurisdictional claims in published maps and institutional affiliations.

## Supplementary Material

Supporting Information
