# Supplementary material for: Unidirectional invisibility induced by parity-time symmetric circuit
Source: Sci Rep. 2017 Jan 18;7:40575. doi: 10.1038/srep40575 (PMC5241775; doi:10.1038/srep40575)
Supplement: Supporting Information [file srep40575-s1.pdf]

# Support information of "Unidirectional invisibility induced by parity-time symmetric circuit"

Bo Lv[1], Jiahui Fu[1*] , Bian Wu[2], Rujiang Li[3], Qingsheng Zeng[4], Xinhua Yin[5], Qun Wu, Lei Gao[4], Wan Chen[1], Zhefei Wang[1], Zhiming Liang[1], Ao Li[1], Ruyu Ma[1]

[1]*Microwave and Electromagnetic Laboratory, Harbin Institute of Technology, No.92, Xidazhi Street, Nangang District, Harbin City, Heilongjiang Province, China*

[2]*School of Electronic Engineering, Xidian University, Xi'an, 710071, China*

[3]*College of Information Science and Electronic Engineering, Zhejiang University, Hangzhou 310027, China*

[4]*Propagation Group, Wireless Technologies Branch, Communications Research Centre Canada, Government of Canada, 3701 Carling Ave., Box 11490, Station H, Ottawa, Ontario K2H 8S2, Canada*

[5]*Harbin medical university, No.157, Baojian Street, Nangang District, Harbin City, Heilongjiang Province, China*

** is the corresponding author*

*fjh@hit.edu.cn*

**Supplementary Note 1**

**Scattering matrix derivation for the proposed PT-symmetric device.**

We generally consider two parallel lumped resistors $R_1$ and $R_2$ separated by two portions of lossless transmission-line of electric length $l_1 = kd_1, l_2 = kd_2$ and the characteristic impedance is $Z_0$. The reactance component $X = 1/\omega C$ or $X = \omega L$ between the two transmission lines is the impedance of the capacitor $C$ or the inductor $L$. We note $r_1, r_2$ and $x$ the normalized quantities $r_1 = R_1 / Z_0, r_2 = R_2 / Z_0$ and $x = X / Z_0$. Here the input impedance is consistent with the characteristic impedance that is $R_1 = Z_0$ and $r_1 = 1$ for the incident-energy reflectless. In the electric circuit, we construct the electric PT-symmetric conformation, thus the relation between the two resistors presents $r_1 = -r_2 = 1$. Therefore, the normalized transmission matrix of this two-port system is calculated by cascading the normalized transmission matrices of all its sub-units under an $e^{j\omega t}$ time evolution [1]

$$M=\begin{pmatrix}1 & 0\\ 1 & 1\end{pmatrix}\cdot\begin{pmatrix}\cos l_1 & j\sin l_1\\ j\sin l_1 & \cos l_1\end{pmatrix}\cdot\begin{pmatrix}1 & 0\\ 1/jx & 1\end{pmatrix}\cdot\begin{pmatrix}\cos l_1 & j\sin l_1\\ j\sin l_1 & \cos l_1\end{pmatrix}\cdot\begin{pmatrix}1 & 0\\ -1 & 1\end{pmatrix}=\begin{pmatrix}M_{11} & M_{12}\\ M_{21} & M_{22}\end{pmatrix} \tag{1}$$

with

$$M_{11}=-(j\cos l_1+\sin l_2)(j\sin l_1+xj\cos l_1+x\sin l_1)/x$$

$$M_{12}=j\sin l_2(\cos l_1+(\sin l_2)/x)+j\sin l_1\cos(l_2)$$

$$M_{21}=-(j\cos(l_1-l_2)-\sin(l_1-l_2))/x$$

$$M_{22}=j(\cos l_1+j\sin l_1)(j\sin l_2+jx\cos l_2-x\sin l_2)/x$$

From the transmission matrix *M*, we calculate the scattering matrix S [1]:

$$S=\begin{pmatrix}S_{11} & S_{12}\\ S_{21} & S_{22}\end{pmatrix}=\begin{pmatrix}\dfrac{M_{11}+M_{12}-M_{21}-M_{22}}{M_{11}+M_{12}+M_{21}+M_{22}} & \dfrac{2\det(M)}{M_{11}+M_{12}+M_{21}+M_{22}}\\ \dfrac{2}{M_{11}+M_{12}+M_{21}+M_{22}} & \dfrac{M_{22}+M_{12}-M_{21}-M_{12}}{M_{11}+M_{12}+M_{21}+M_{22}}\end{pmatrix} \tag{2}$$

We get the formation of *S* matrix:

$$S_{11}=\frac{j\cos l_1\cos l_2-jx\sin(l_1+l_2)}{2\sin l_1\cos l_2-j\cos l_1\cos l_2+2x\cos(l_1+l_2)+jx\sin(l_1+l_2)} \tag{3}$$

$$S_{12}=S_{21}=\frac{2x}{2\sin l_1\cos l_2-j\cos l_1\cos l_2+2x\cos(l_1+l_2)+jx\sin(l_1+l_2)} \tag{4}$$

$$S_{22}=\frac{-3j\cos(l_1+l_2)+5j\cos(l_1-l_2)-4\sin(l_1-l_2)+6jx\sin(l_1+l_2)}{2(2\sin l_1\cos l_2-j\cos l_1\cos l_2+2x\cos(l_1+l_2)+jx\sin(l_1+l_2))} \tag{5}$$

Such a matrix indeed fulfills the special symmetry $PTS(\omega^*)PT=S^{-1}(\omega)$, as expected in PT-symmetric systems [2].

**Supplementary Note 2**

**Unidirectional reflectionless condition**

Independently of the reactance component, the case $l_1, l_2=\pi/2+N\pi$ where *N* is integer yields the unidirectional reflectionless condition:

$$S=\begin{pmatrix}0 & \pm 1\\ \pm 1 & \pm j2/x\end{pmatrix} \tag{6}$$

where the $\pm$ signs of $S_{12}$, $S_{21}$ and $S_{22}$ depend on the integer *N* being odd or even respectively. This case corresponds to reflectless from input and the complete transmission presents in both ports. Furthermore, the reflection from output is non-zero and increasing with lower value of the normalized reactance component *x*. As the analysis in the main article, the electric device presents the unidirectional performance. Because the effect of the active device adjacent to the output, the

reflection $S_{22}$ can be larger than unitary. Additionally, the eigenvalues of the $S$-matrix under the unidirectional reflectionless condition are calculated as

$$\lambda_1, \lambda_2 = \frac{j \pm \sqrt{x^2 - 1}}{x} \tag{7}$$

where the signs of $S_{12}$, $S_{21}$ and $S_{22}$ is positive, and the eigenvalues can changed the sign in contrary sign of S-matrix. For different values of $x$, we get the relation $|\lambda_1 \lambda_2| = 1$. When the resistance value of the reactance component is lower than characteristic impedance that is $x < 1$, the eigenvalues present pure imaginary. Furthermore, the magnitude of reflection from the output $|S_{22}| > 2$ presents the well-defined unidirectional performance. When the resistance value of reactance component is equal to the characteristic impedance that presents $x = 1$, the eigenvalues presents the formation that is $|\lambda_1| = |\lambda_2| = 1$ which correspond to the spontaneous PT-symmetry breaking point, and the magnitude of $|S_{22}| = 2$. For the case $x > 1$ that is the resistance value of reactance component is higher than the characteristic impedance, the eigenvalues of S-matrix present complex number, and the magnitude of reflection from the output $|S_{22}| < 2$ presents the poorly unidirectional performance [3]. Based on the above analysis, the electric system presents the unidirectional function by lower resistance value of reactance component.

**Supplementary Note 3**

**The design of the PT-symmetric circuit**

For DC-blocking the external-input/output energy, we add the capacitors $C_a = 1\mu F$ at the input and output in the main thread of the circuit. The normalized quantities of the capacitor at the frequency $f$ = 2 GHz satisfies the relation $r_{C_a} = (1/j\omega C_a)/Z_0 \to 0$, where the angular frequency $\omega = 2\pi f$. Further more, the capacitors $C_a$ is mathematically equivalent to the series reactance matrix at front and behind end of the transmission matrix $M$:

$$M_a = \begin{pmatrix} 1 & r_{C_a} \\ 0 & 1 \end{pmatrix} \cdot \begin{pmatrix} 1 & 0 \\ 1 & 1 \end{pmatrix} \cdot \begin{pmatrix} \cos l_1 & j\sin l_1 \\ j\sin l_1 & \cos l_1 \end{pmatrix} \cdot \begin{pmatrix} 1 & 0 \\ 1/jx & 1 \end{pmatrix} \cdot \begin{pmatrix} \cos l_1 & j\sin l_1 \\ j\sin l_1 & \cos l_1 \end{pmatrix} \cdot \begin{pmatrix} 1 & 0 \\ -1 & 1 \end{pmatrix} \cdot \begin{pmatrix} 1 & r_{C_a} \\ 0 & 1 \end{pmatrix} \tag{8}$$

In Eq. (8), the added matrix is the function of the identity matrix approximatively when $r_{C_a} \to 0$ and the transmission matrix $M_a \to M$ at the frequency $f$. Thus, the

added capacitor $C_a$ has little effect on the AC state of the circuit and blocking the DC-input/output energy effectively.

**Supplementary References**

1. D. M. Pozar, *Microwave Engineering* (Wiley, 2011).

2. Y. D. Chong, L. Ge & A. D. Stone, PT-Symmetry Breaking and Laser-Absorber Modes in Optical Scattering Systems, Phys. Rev. Lett. 106 093902 (2012).

3. Y. Sun, W. Tan, H. Li, J. Li & H. Chen, Experimental Demonstration of a Coherent Perfect Absorber with PT Phase Transition, Phys. Rev. Lett. 112, 143903 (2014).
